# Supplementary material for: Individual and community level factors associated with modern contraceptive utilization among women in Ethiopia: Multilevel modeling analysis
Source: PLoS One. 2024 May 16;19(5):e0303803. doi: 10.1371/journal.pone.0303803 (PMC11098393; doi:10.1371/journal.pone.0303803)
Supplement: Attachment — Submitted filename: Review II.pdf [file pone.0303803.s002.pdf]

Manuscript Number: PONE-D-23-09872

Title: Individual and community level factors associated with modern contraceptive use among women in Ethiopia: Multilevel modelling analysis

Version: 2 **Date:** 12 August 2023

Major comment

If you correct the comment given to you based on your research article study accordingly, it will be considered for publication.

Abstract

- Your abstract too shallow especially in background and conclusion part.
- What about other finding and factors associated with modern contraceptive methods like socio-demographic status, fertility status
- Your finding is not matched with your study protocol which it is minimized
- Your conclusion in the abstract is not based on your findings.

Background

- Your background is too wide you should have minimized based on journal guidelines.
- In paragraph 1 you have explained only married women what about unmarried but uses family planning based on your title.
- Your first paragraph should be defined the problem or subject matter rather than general term.
- Then the next paragraphs start to problem based idea and its prevalence, burden, consequence, strategies to overcome and complication from global to local.
- Your background not talk about general facts of your study outcome variables and to shallow, add as comment given to in your document.
- Your back ground amalgamated terminologies and it needs revision one by one.
- Most of your document lacks the references and need updating at least five year duration.
- If you are using secondary data, you doesn't mention about data extraction period.
- Some of idea in your document it lacks clarity, typographical errors especially grammar how did you access it.
- Justification /rationale of the study is not clear. You have to clearly state why this study was necessary in concise and coherent way. ***It needs critical revision.***

Methods

- How you see the difference of study population and sampled population? Correct it.
- Focus only on the objective of the study.
- EDHS 2019 was mini EDHS and have you accessed all variables what you want to analyze?
- In Ethiopia each region had multi diversified populations, cultural, political, religious etc. how did you justified the sampling methods and stratification methods urban or rural, large region or small region. It is difficult to estimate and generalize based on your way of interpretation and sampling methods. ***It needs critical revision.***

- Inclusion and exclusion criteria, dependent variable and independent variables should be clearly justified. ***<u>It needs also critical revision.</u>***
- What are the unknown confounding factors of this study finding and think your study design?

Result

- How did you categorized age category, why not based on WHO category or EDHS it self category? Is it inclusive? Because majority of rural part of community doesn't know their real date of birth and ***<u>it needs critical revision.</u>***
- Please carefully cross-check the numbers (percentages) in the texts and respective tables (E.g.Table 1(No education 2600 but Literacy for those with no education (2589), wealth index, total live birth etc.), please revise and put denominator in each part of table. ***<u>It needs also critical revision.</u>***
- Is it homogenies which means cluster? What are non-cluster parts of study populations?

Discussion

- Your discussion part should be rewritten. Please focus only in line with your important findings and objectives of the study.
- Your first paragraph should be you magnitude and objective of your study then factors associated with.
- Factors like the marital status, education status of women is it analyzed and output of your study? How did you accessed and from where? Not found in table.
- Recommendation and strong conclusion should be needed.
- Limitation of study needed.
- This all are critical part of your study and you should have revise it

I am not comfortable with this study finding and justification, unless you come up with strong justification, the idea is novel and what make your study stronger the only reason.

Thank you!
