# Supplementary material for: Individual and community level factors associated with modern contraceptive utilization among women in Ethiopia: Multilevel modeling analysis
Source: PLoS One. 2024 May 16;19(5):e0303803. doi: 10.1371/journal.pone.0303803 (PMC11098393; doi:10.1371/journal.pone.0303803)
Supplement: Attachment — Submitted filename: Ethiopia paper Manuscrpit_April_2_23 (1).docx [file pone.0303803.s003.docx]

Individual and community level factors associated with modern contraceptive use among women in Ethiopia: Multilevel modelling analysis

Hailay Gebremichael Gebrekidan^1,2*^, Mussie Alemayehu^1^, Gurmesa Tura Debelew^2^

^1^Mekelle University, College of Health Sciences, Mekelle, Ethiopia

^2^Department of population and family health, Institute of Health, Jimma University, Jimma, Ethiopia

* Corresponding Author

POB: 378

Mobile: -+251-966-346-756

HGG: [hailaygeb23@gmail.com](mailto:hailaygeb23@gmail.com)

Email address

GTD: [gurmesatura@gmail.com](mailto:gurmesatura@gmail.com)

MA: [mossalex75@gmail.com](mailto:mossalex75@gmail.com)

# Abstract

**Back Ground:** Modern contraceptive use reduces maternal and child mortality by reducing unplanned pregnancies. In addition, contraception has a demographic and human rights rationale. Despite these benefits, in Ethiopia, the utilization of modern methods of contraceptives is low and far away from the national target. Therefore, understanding the underlying caveats for contraceptive use is critical to addressing low contraceptive use.

**Objective**: To identify modern contraceptive use and factors associated with modern contraceptive use among women in Ethiopia.

**Methods:** This study included data from a national community-based, cross-sectional survey of the performance monitoring for action conducted in Ethiopia in October 2019. A total of 6,117 women aged 15-49 years, a subset of the largest national family planning (FP) study, were included in the analysis. The outcome measure of the study was self-reported modern contraceptive use or non-use. Data were analyzed using R software version 4.1. A two-level mixed-effects logistic regression model was used to estimate the associated factors on modern contraceptive use. The results were described using the adjusted odds ratio.

**Results:** The prevalence of modern contraceptive use was 34.5% (33.3 to 35.7). Women with primary, secondary, and above secondary school attendance were more likely to report modern contraceptive use with AOR of 1.47, 1.73, and 1.58, respectively. Information exchange about FP at the facility was positively associated with contraceptive use (AOR:1.84, 95% CI 1.52, 2.23). Community differences also played some role in contributing to contraceptive use, to which 21.9% of the total variance in the odds of using modern contraceptives was attributed (ICC = 0.219). Clusters with a higher proportion of agrarian (AOR: 2.27, 95% CI 1.5, 3.44), clusters with higher literacy (AOR: 1.46, 95% CI 1.09, 1.94), clusters with empowered women and girls about FP (AOR: 1.47, 95% CI 1.11, 1.93) and clusters with high supportive attitudes and norms toward FP (AOR: 1.37, 95% CI 1.04, 1.81) had better modern contraceptive use than their counterparts.

Conclusions: In Ethiopia, modern contraceptive use in women is influenced by the individual- and community-level factors. Intervention programs and strategies to increase contraceptive use should target women, involve communities, and be tailored to cluster characteristics and ways of living.

Keywords: Modern contraceptive methods, Multilevel, Interval odds ratio, Ethiopia, Median Odds ratio,

# Introduction

Family planning enables couples and individuals to realize their fundamental right to decide freely and responsibly the number and spacing of their children (1,2). The International Conference on Population and Development (ICPD) gave due emphasis on the basic right of all couples and individuals to decide freely and responsibly on the number of children to have by spacing and timing of their children and to have the full information and means to do so (1). Along with this, family planning has demographic, health, and human rights rationale (3).

Globally, in 2021, 45 percent of married or in-union women of reproductive age were using modern contraceptives. However, in Sub-Saharan Africa, modern contraceptive use was much lower (4). Many of today’s poorest countries, mainly in sub-Saharan Africa, still have high fertility and a high unmet need for family planning, and their populations are projected to double in the next few decades (5).

Overall in Ethiopia, 41% of currently married women are using modern methods of family planning, which is 14% less than the target of 55% (6,7). The London Summit of FP2020 was launched to improve contraception uptake and targeted 120 million additional women and girls in 69 of the world’s poorest countries by 2020 (8). In line with the FP2020, Ethiopia committed to reaching an additional 6.2 million women with FP services and increasing CPR to 55% by 2020 (9). However, the 2016 progress report of the 69 countries showed that countries were off track to achieving the overall goal of FP2020 (10).

Globally, unintended pregnancy and births are becoming a major public health problem that causes illness during pregnancy, complications at birth, and maternal deaths (11). Considerably, unintended pregnancy and birth are higher in developing regions than in developed regions (11,12).

In countries with high birth rates, family planning can mainly reduce maternal and child mortality by reducing unplanned pregnancies (13). Besides, it is highly cost-effective in poverty reduction and helps women achieve their human rights to health, autonomy, and personal decision-making about family size (14). A fact sheet report indicated that if all unmet needs for modern contraception were satisfied in Ethiopia, unintended pregnancies would drop by 90%, from 2.1 million to 219,000 annually. As a result, the annual number of unplanned births would decrease from 1.2 million to 128,000, and the number of abortions would drop from 571,000 to 59,000 (15).

So far, Ethiopia has been working to improve contraception uptake and maternal, neonatal, and child health. It implemented the first Health Sector Transformation Plan (HSTP-I) from 2015/16 to 2019/20. Several positive results and achievements were marked in decreasing maternal and child mortality and increasing contraception utilization (7). Besides, Ethiopia’s commitment to increasing contraceptive prevalence in the FP2020 program was another effort (16).

Despite the efforts of the government and NGOs and the immense benefits of contraceptives, in Ethiopia, the utilization of modern methods of contraceptives is lower, and the unmet contraceptive need has increased to 22% (7). Generally, the country is behind the global family planning goals and targets, despite all these efforts made and specific signs of progress counted (9).

However, Ethiopia has been working to ensure continuity and improve the momentum of achieving the HSTP II ambitious targets to reduce the maternal mortality rate to 279 per 100,000 live births and to reduce under-5 and neonatal mortalities to 44 and 21 per 1,000 live births and the Sustainable Development Goals (SDGs), target 3.7, which calls on countries “by 2030, to ensure access to family planning, information, and education (7,17).

Hence, understanding the underlying caveats for contraceptive use is critical to addressing low contraceptive use in the country. Several studies elsewhere in the country revealed many reasons for low contraceptive use. However, most are small, lack depth, identify solely individual factors without consideration of the contextual factors, and have substantial heterogeneity impeding a robust conclusion. Moreover, it is not always for granted to consider factors that are important in one area and would also be so in others due to differences in contexts, culture, beliefs, community structure, and way of life. Besides, they fail to determine whether the environment or community plays a positive or negative role in contraceptive use. This is particularly important in multi-ethnic and multicultural countries such as Ethiopia. Also, the communities in Ethiopia live dispersed, some with difficult access and others with an extreme way of living, ranging from urban to agrarian and nomadic communities.

Changes in the international family planning field have now refocused attention on all women. Despite being prone to unwanted pregnancy, unmarried women are often excluded and broadly underrepresented in most studies. In addition, most prior evidence about contraceptive use has typically been produced for married/in-union women who missed considering unmarried women. A report indicates that measuring, monitoring, and reporting MCPR for all women of reproductive age reinforces the right of all women to use contraception (18). So, this current study was done for all reproductive-age women who could be considered the best representative of the target population.

It is just crucial to assess all the underlying reasons and contextual challenges to tackle them and achieve the target goals for contraceptive prevalence and, thus, the benefits. Hence, this study aimed to examine factors (individual and community-level characteristics and between-community differences) on modern contraceptive use. Past multilevel analysis results were described by only odds ratio, the proportion of change in cluster variation (PCV), and intraclass correlation (ICC). But this current study considered summary measures of the effect of cluster variables (Interval Odds ratio (IOR) and proportion of opposed odds ratio (POOR)) and measures of components heterogeneity MOR (Median odds ratio) to make our result more informative and explanatory.

The result will help guide health policymakers to tailor efficient FP programs to the community by considering individual and community level factors to enhance the low CPR and contribute to equitable access instead of devising a one-size-fits-all strategy that may work better in one community but not in others.

# Objective

The objective of this study is to assess the factors that influences (community and individual factors on a woman’s likelihood of ) using a modern contraceptive among women in Ethiopia.

# Methods and Materials

## Study Design and data source

We used data from Ethiopia’s national performance monitoring for action (PMA) cross-sectional survey in 2019 that focused on reproductive, maternal, and newborn health, including FP (see details on the program at https://www.pmadata.org/countries/ethiopia). The survey covered all regions, sample subregions and communities, and individual women. For the current study, we took subset data from the national survey pertinent to the research.

The sampling of regions (the largest administrative divisions), subregions and communities (or enumeration areas [EA]), and individual women was designed centrally by Ethiopia’s statistical agency, ensuing representativeness at the national and sub-regional levels for both urban and rural areas. EAs were drawn with probability proportional to size (PPS) within the strata. For some of the larger regions (Tigray, Amhara, Oromia, SNNP), EAs were drawn separately for urban and rural strata within the region. For Afar, Somali, Benishangul-Gumuz (BG), Gambella, and Harari, the EAs were randomly selected with PPS in the regions— i.e., without urban and rural stratification. Addis Ababa is exclusively urban, so no stratification was used. In the six large regions, a census of the households within the selected EAs was conducted, listing the names, sex, and ages of all household members. This served as a sampling frame to randomly select 35 households per EA. Complete household listing was conducted in the remaining regions, from which 35 households were randomly selected. Finally, all women of reproductive age 15 to 49 within the selected households were eligible for interviews. Data on households and individual women of reproductive age were collected by trained enumerators who received intensive training and were overseen by supervisors. Surveys were developed on ODK, open-source software for collecting and managing data, and loaded on smartphones. The data were sent at the point of the interview to a central server, where they were quality checked, allowing the enumerators to correct any errors that occurred without delay. The details of the PMA-Ethiopia survey design and sampling techniques are described elsewhere (19). A two-level multilevel logistic regression analysis was used to examine the effects of individual and community-level characteristics and between-community differences on modern contraceptive use.

## Sample

For the current study, 6,117 women who consented to participate, who were not pregnant, fecund, and sexually active, were finally included in the analyses (figure 1).

## Variables

This study used a two-level random intercept logistic regression model to estimate the effect of unobserved characteristics of the cluster of respondents on the likelihood of modern contraceptive use. The outcome variable in this study was modern contraceptive method use/non-use. So, the independent variables were categorized into the level I (individual women variables) and level II (cluster/community level variables) when we say cluster level II (community-level variables).

#### Individual-level variables

The level 1 (individual women level) variables included age, educational status, marital status, marriage history, place of birth, parity, Educational Status, religion, the intention when to have the next birth, household wealth quintile, knowledge of contraceptive methods, whether visited a health facility in the past 12 months and discussed family planning, whether visited by a health worker in the past 12 months (Table 1).

#### Community-level variables

Community-level variables are created by aggregating the individual-level variables. Hence, based on the mean/median value of the response, the variable labeled as “lower” and “higher” categories for the cluster that scored below mean, mean, and above mean value, respectively (Table 1).

Table 1: Descriptions of independent variables considered in this study

| **Variables** | **Descriptions** | **Measurements** |
| --- | --- | --- |
| **Dependent variable** | | |
| Modern contraceptive use | The utilization of modern contraceptives in reproductive-age women at the time of the survey. | A woman who was using any one of the modern contraceptive methods during the survey time was coded as “1” and who was not used as “0” |
| **Independent level variables** | | |
| Age | Age of women in complete years at the interview time | Categorized for analysis into 15-19, 20-34, and 35-49 years. |
| Educational Status | The highest level of education a woman has attained | Categorized into four groups “No education”, “Primary”, “secondary”, and “More than secondary”. |
| Marital status | Marital status of women at the interview time | Categorized into groups “Never married”, “Married or in union/relationship”, and “Divorced/widowed”. |
| Visited by the health worker | A woman was visited by HEW who talked about FP in the past 12 months | A woman who was visited by HEW who talked about FP in the past 12 months was coded as “1” and was not as “0” |
| Wealth quintiles | Household assets, home, land, and livestock ownership were assessed, and the wealth index was computed by using principal component analysis (PCA) | The wealth status was categorized into five groups “Lowest quantile”, “Lower quantile”, “Middle quantile”, “Higher quantile”, and “Highest quantile”. |
| **Community level Variables** | | |
| Women and girls empowerment about FP | The women were asked to respond to five empowerment-related questions. | Accordingly, as a result was normally distributed, the mean score was computed for the women and girls empowerment about FP, and it was categorized those below the mean as “Not Empowered”, and mean and above the mean as “Empowered” |
| Exposure to family planning information | The respondents were asked five exposure to family planning information-related questions. E.g. In the last few months have you heard about family planning on the radio?? The response was “yes” or “no”. Then its response summed up to form one continuous variable. | Accordingly, as a result, was normally distributed the mean score was computed for exposure to family planning information and it was categorized below the mean as “Lower exposure”, and mean and above the mean as “higher exposure” |
| Community way of life | Community way of life was categorized into pastoralist and based on the Ethiopian regions category. Based on that, regions 1, 3, 4, 7, 9, 10, and 11 were categorized as agrarian, and regions 2, 5, 6, and 8 were categorized as pastoralist communities. | Hence, the communities were categorized into two “Pastoralist” and “agrarian” |
| Community attitude/norms towards contraceptives | The women were asked to respond to five community attitudes/norms towards family planning questions. Some of the items were “Women who use family planning are considered promiscuous”. The response was scored as “strongly agree”, “somewhat agree”, “Neither agrees nor disagree”, “somewhat disagree”, and “strongly disagree”. Items negatively worded were reversed and scored. Then, the response is summed up to generate a continuous variable. | Accordingly, as a result was normally distributed, the mean score was computed for Community norms about FP, and it was categorized below the mean as “Non-supportive norm”, and mean and above the mean as “Supportive norm” |
| Community knowledge of the modern contraceptive method | The women were asked whether they had heard of each of the 11 modern methods of contraceptives, and a composite knowledge score was produced using PCA. The response to 11 items was labeled “yes” and “no”. Examples of items include: “ever heard female sterilization”. | We used the median score as a cutoff point to categorize the composite knowledge. The result was not normally distributed. So, the response was categorized as having “Good knowledge of modern contraceptives” for those who scored the median and above the median and “Poor knowledge of modern contraceptives” for those who scored below the median. |
| Community literacy level | It was measured by asking the respondents about the highest school they attended and categorized into “lower literacy” and ‘higher literacy.” | Accordingly, as a result, the mean score was normally distributed for the community literacy level, and it was categorized below the mean as “lower literacy”, and mean and above the mean as “higher literacy.” |

# Statistical Analysis

The data obtained from the PMA official database was exported and analyzed by R software version 4.1.3. In the initial step, to clean and prepare the data for the principal analysis, exploratory data analysis, including extracting, coding, and recoding, categorizing continuous variables, and constructing aggregate community-level variables from individual-level variables, was done by Stata version 16.1. Descriptive statistics were performed by determining the frequency distributions and percentages along the different individual reproductive age women and community level variables.

Since the reproductive age women are nested within cluster/EA, a two-level mixed-effect logistic regression model was employed to describe the individual and community-level variables within and between cluster effects on current modern contraceptive use. Four models were fitted, consisting of the empty model I, Model II, Model III, and Model IV. Multivariate two-level logistic regression analysis was performed to estimate the adjusted odds ratio (AOR) for individual and community characteristics and the extent of random variations between communities.

**Model I (Empty model):** We run the model without explanatory variables. This aimed to test the random effect of the between-cluster variability. Derived from the between and within-cluster variability, the intra-class correlation (ICC) was estimated to explain if the data justified using a multilevel approach for analyses by depicting the level of variability between clusters.

Model II: Examined the effects of individual-level variables on modern contraceptive use. Statistically significant individual-level variables in the bivariate multilevel logistic regression analysis were included in this model.

Model III: Examined the effect of community-level variables on modern contraceptive use. Those community-level variables, which were statistically significant in the bivariate multilevel logistic regression analysis, were included in this model. They were formed by aggregating the variable from the individual-level variables.

**Model IV:** The fourth step considered a model building for two multiple multilevel logistic regression analysis levels. Those variables statistically significant in model II and model III were included in this model. This final model identified average associations between specific individual characteristics and community-level variables at the same time while adjusting for clustering (20).

The multilevel analysis of individual heterogeneity approach combines both the multilevel analysis of associations for the estimation of specific contextual effects and the multilevel analysis of variance (e.g., the degree of clustering, ICC) for the investigation of general contextual effects (i.e., non-specific contextual influences on contraceptive use/non-use (21,22).

For cluster-level variables, the concept of an intra-cluster association is not as straightforward as in the case of individual-level variables because the value of cluster-level variables is constant for all individuals in the cluster. Therefore, one needs to consider assuming the existence of several clusters. In addition, various alternatives to the ICC for binary responses have been proposed to quantify the extent of general contextual effects. These include Shrinkage factor, IOR (Interval odds ratio), POOR (proportional opposed odds ratio), MOR (Median Odds ratio), and PCV (variance partition coefficient).

Shrinkage factors are used to calculate interval odds ratio (IOR) and proportion of opposed odds ratio (POOR), which are summary measures of the effects of cluster-level variables. On the model considering the conditional or cluster-specific interpretation of the regression coefficient, a shrinkage factor was calculated to calculate the marginal or population average regression coefficient and odds ratio. As the shrinkage factors are very close to one, the population-average regression coefficients (and corresponding population-average odds ratios) will be very close to the conditional or cluster-specific regression coefficients and corresponding odds ratios (23,24). This eases the interpretation of an association between the cluster-level variable and the outcome variable.

The IOR is a fixed-effects measure for quantification of the effect of cluster-level variables. It is defined as an interval for odds ratios covering the middle 80 percent of the odds ratios of different cluster-level covariates or the middle 80% range of the distribution of ORs formed by randomly comparing clusters exposed and non-exposed to the contextual variable. The interval is narrow if the between-cluster variation is small and wide if the between-cluster variation is large. If the interval contains 1, the cluster variability is large compared to the effect of the cluster-level variable. If the interval does not contain 1, the effect of the cluster-level variable is large compared to the unexplained between-cluster variation (25,26). According to the model, the odds ratios can take any value between zero and infinity. However, to put meaning into the IOR, it is natural to report an interval likely to contain the odds ratio when randomly choosing two persons with specific covariates (25).

An alternative to the IOR-80% is the Proportion of Opposed Odds Ratios (POOR). That is the proportion of ORs in the opposite direction to the overall OR. The values of the POOR extend between 0% and 50%. A POOR of 0% means all ORs have the same sign. A POOR of 50% means that half of the ORs are of the opposite sign, so the association is very heterogeneous (26).

The estimated coefficients and odds ratios IOR and POOR above do not provide a measure of general contextual effects. So, we need to have a measure of components of variance (the variance partition coefficient (VPC) or intra-class correlation (ICC) and Median Odds ratio (MOR) for variation or heterogeneity in outcomes between clusters. VPC/ICC represents the proportion of the total observed individual variation in the outcome attributable to between-cluster variation. It measures the proportion of total variance due to the difference between groups. The higher this proportion, the higher the general contextual effect. The VPC/ICC is a key concept in the multilevel analysis as it quantifies the proportion of observed variation in the outcome attributable to the clustering effect (27).

The VPC (ICC) measures components of variance (clustering) that considers both between- and within-cluster variance. The VPC value ranges from zero to one; if all subjects in the same cluster exhibited the same response, it has a value of 1, and it would be 0 if there were no within-cluster homogeneity of responses (23). In line with this, a proportional change in cluster variation (PCV) is calculated for all models by taking the empty model as a reference point. Percentage Change in Variance (PCV) indicates the proportion of the total observed individual variation of modern contraceptive utilization attributable to cluster variations_._ The MOR is a measure of heterogeneity mainly dependent on between-cluster variance. It revealed the median value of the odds ratio between the cluster at high modern contraceptive utilization and the cluster at low modern contraceptive utilization when randomly picking two individuals with the same covariate values from two different clusters (28). The MOR is the median odds ratio between a person of higher and lower propensity. Therefore, the higher the MOR, the greater the general contextual effect (29). The measure is always greater than or equal to 1. If the MOR is 1, there is no variation between clusters (no second-level variation). If there is considerable between-cluster variation, the MOR will be large. The measure is directly comparable with fixed-effects odds ratios (25).

Multi-Co linearity among the independent variables was checked using the tolerance and the Variance Inflation Factor (VIF). Model comparison was made using Akaike’s Information Criterion (AIC) tests. The model with the lowest value of AIC was considered a better-fit model. Adjusted odds ratios and associated 95% confidence intervals (CIs) were presented for all three models except model I.

# Ethical consideration

Ethical approval was not required since we have used publicly available PMA-Ethiopia datasets. We have accessed the data set by online application submitted to Johns Hopkins School of Public Health through [www.pmadata.org/countries/ethiopia](http://www.pmadata.org/countries/ethiopia) after telling the purpose of the study.

# Results

## Individual-level variables

This study focused on a sample of 265 clusters in Ethiopia. We included 6117 reproductive-age women in the 265 clusters. Around 2600(42.5%) of the respondents were with no education. Of these, 2489(96%) could not read and write a simple sentence. More than half, 3487(57%) of the respondents were within the age category of 20-34. About 4885(79.8%) of the respondents were married/living with a partner. About 1179 (22.2%) respondents had six or more children. The proportion of respondents who preferred wait duration from now before another child's birth was 2616(65.2%). Modern contraceptive use among the respondents was 34.5% (33.3-35.7). However, 97.3% of the respondents have heard of at least one modern contraceptive method. About 1548 (42%) of non-current users of contraceptives have a future intention to use (Table 2).

Table 2: Selected individual-level variables of respondents, Ethiopia, PMA, 2019

| **Individual Characteristics** | **N** | **%** | **95% CI** | |
| --- | --- | --- | --- | --- |
| Educational status of women |  |  |  |  |
| No education | 2600 | 42.5 | 41.27 | 43.75 |
| Primary | 2034 | 33.25 | 32.08 | 34.4 |
| secondary | 883 | 14.4 | 13.57 | 15.3 |
| More than secondary | 600 | 9.8 | 9.08 | 10.57 |
| Literacy for those with no education |  |  |  |  |
| No | 2489 | 95.9 | 95 | 96.5 |
| Yes | 107 | 4.1 | 3.4 | 4.9 |
| Birthplace |  |  |  |  |
| urban | 1027 | 16.78 | 15.87 | 17.74 |
| Rural | 5090 | 83.2 | 82.25 | 84.1 |
| Religion |  |  |  |  |
| Orthodox Christian | 3008 | 49.1 | 47.9 | 50.4 |
| Muslim | 1747 | 28.5 | 27.4 | 29.7 |
| Protestant | 1230 | 20.1 | 19.1 | 21.1 |
| other | 132 | 2.1 | 1.8 | 2.5 |
| Age |  |  |  |  |
| 15-19 | 444 | 7.25 | 6.6 | 7.93 |
| 20-34 | 3487 | 57 | 55.7 | 58.2 |
| 35-49 | 2186 | 35.74 | 34.5 | 36.9 |
| Marital Status |  |  |  |  |
| Never married | 363 | 5.9 | 5.37 | 6.55 |
| Married or in a union/relationship | 4885 | 79.8 | 78.8 | 80.8 |
| Divorced/widowed/widower | 869 | 14.2 | 13.3 | 15.1 |
| Wealth Index quantile |  |  |  |  |
| Lowest quintile | 1002 | 16.38 | 15.47 | 17.3 |
| Lower quintile | 1027 | 16.79 | 15.87 | 17.7 |
| Middle quintile | 1027 | 16.79 | 15.87 | 17.7 |
| Higher quintile | 1239 | 20.25 | 19.26 | 21.28 |
| Highest quintile | 1821 | 29.77 | 28.64 | 30.9 |
| Total live birth |  |  |  |  |
| 1 | 1184 | 22.6 | 21.5 | 23.79 |
| 2-3 | 1739 | 33.2 | 31.9 | 34.5 |
| 4-5 | 1128 | 21.5 | 20.47 | 22.7 |
| 6-14 | 1179 | 22.5 | 21.4 | 23.69 |
| Sex at the first age |  |  |  |  |
| 9-15 | 2183 | 35.68 | 34.49 | 36.9 |
| 16-24 | 3550 | 58 | 56.8 | 59.2 |
| 25-39 | 384 | 6.27 | 5.7 | 6.9 |
| Ever been pregnant |  |  |  |  |
| no | 814 | 13.3 | 12.48 | 14.18 |
| yes | 5303 | 86.7 | 85.8 | 87.5 |
| Was visited by HEW, who talked about FP in the past 12 months |  |  |  |  |
| no | 5362 | 87.7 | 86.8 | 88.4 |
| yes | 755 | 12.3 | 11.5 | 13.2 |
| Attended a group FP counseling session with a provider in the past 12 months |  |  |  |  |
| no | 5459 | 89.2 | 88.4 | 90 |
| yes | 658 | 10.8 | 10 | 11.5 |
| Staff spoke about FP methods at the facility visit |  |  |  |  |
| no | 2,872 | 76.4 | 75 | 77.7 |
| yes | 889 | 23.6 | 22.3 | 25 |
| Will you use family planning in the future (not current users) |  |  |  |  |
| No | 2139 | 58 | 56.4 | 59.6 |
| Yes | 1548 | 42 | 40.4 | 43.6 |
| Current modern contraceptive use |  |  |  |  |
| No | 4007 | 65.5 | 64.3 | 66.7 |
| Yes | 2110 | 34.5 | 33.3 | 35.7 |
| All contraceptive utilization |  |  |  |  |
| No | 3921 | 64.1 | 62.8 | 65. 3 |
| Yes | 2196 | 35.9 | 34.7 | 37 |

## Community-level variables

Two hundred sixty-five clusters were considered as a unit of analysis. Accordingly, 52% and 59.6% of the clusters had lower community literacy levels and exposure to FP information, respectively. Above half (52.8%) of the clusters have empowered women and girls about FP. Half (49.5%) of the clusters have a non-supportive attitude/norm toward family planning. Above half (54.2%) of the clusters had poor knowledge of modern contraceptive methods, and 15.2% of the clusters' community way of life belonged to pastoralist communities (Table 3).

Table 3: Selected community-level variables of respondents, Ethiopia, PMA, 2019

| **Community Characteristics** | **No women (%)** | **95% CI** | | **No of clusters** |
| --- | --- | --- | --- | --- |
| Community literacy level |  |  |  |  |
| lower | 3181 (52) | 50.7 | 53.2 | 138 |
| higher | 2936 (48) | 46.7 | 49.2 | 127 |
| Community way of life |  |  |  |  |
| Pastoralist | 927 (15.2) | 14.2 | 16 | 40 |
| Agrarian | 5190 (84.8) | 83.9 | 85.7 | 225 |
| Exposure to FP information |  |  |  |  |
| Low exposure | 3644 (59.6) | 58.3 | 60.8 | 158 |
| High exposure | 2473 (40.4) | 39.2 | 41.6 | 107 |
| Women and girl’s empowerment about FP |  |  |  |  |
| Not empowered | 2889 (47.2) | 46 | 48.4 | 125 |
| empowered | 3228 (52.8) | 51.5 | 54 | 140 |
| Community attitude/norms towards FP |  |  |  |  |
| Non-supportive attitude/norm | 3029 (49.5) | 48.2 | 50.7 | 131 |
| Supportive attitude/norm | 3088 (50.5 | 49.2 | 51.7 | 134 |
| Community knowledge of the modern contraceptive method |  |  |  |  |
| Poor | 3317 (54.2) | 52.9 | 55.4 | 144 |
| Good | 2800 (45.8) | 44.5 | 47 | 121 |

## Multivariable multilevel analyses

## Model building and selection

From the four models Model (I-IV) we fitted, a model with individual and community-level characteristics has the smallest AIC value. Hence, model IV best fits the data. The random effect results were estimated using intra-class correlation (ICC), variance partition coefficient (VPC), a proportional change in variance (PCV), shrinkage factor, Interval odds ratio (IOR), and proportion opposed odds ratio (POOR), Median odds ratio (MOR). Thus to consider all the above, a two-level mixed-effects logistic regression model was considered for further analysis.

As shown in the Table below for the empty model, 21.9% of the total variance in the odds of using modern contraceptives was attributed to the between-cluster variation (ICC = 0.219). The result of the random effect model showed that the intra-class correlation coefficient (ICC) was 21.9%, indicating that the correlation between women in the same EA on modern contraceptive use was 0.219, i.e., 21.9% of the variation in the utilization of modern contraceptives among reproductive age women could be attributed to EA difference.

In the final model, the proportional change of the variance (PCV) of the combined factors was 20.5% (τ=0.732). This indicated that individual and community-level predictors simultaneously explained 20.5% of the variation in utilizing modern contraceptives across communities. Likewise, a shrinkage factor was calculated better to interpret the intra-cluster association in the cluster level factor because the value of the cluster level variable is constant for all individuals in the cluster. The shrinkage factor helps to outfit the conditional interpretation of the regression coefficients with the population average odds ratio. Model 3's shrinkage factor value was 0.893, which is nearly one. This indicates that the population-average regression coefficients (and corresponding population-average odds ratios) for the six clusters/community-level factors are very close to the conditional or cluster-specific regression coefficients and corresponding odds ratios. The 80% Interval odds ratio (IOR) for the six community-level factors in model 3 were (0.48, 10.7) for pastoralist vs. agrarian community way of life and (0. 308, 6.87) for lower vs. higher community literacy level. Besides, it was (0. 31, 6.927) for un-empowered vs. empowered women and girls about FP, (0.29, 6.46) for community non-supportive attitude/norm vs. supportive attitude/norm towards FP. Overall, as the interval contains 1, the cluster variability is large compared to the effect of the cluster-level variable.

In addition, the POOR of the community factors value was 0.248 for the community way of life, 0.378 for the community literacy level, 0.375 for women and girls' empowerment about FP, and 0.397 for community attitude/norms towards FP. Thus, in 37.5% of comparisons between the community with non-empowered and empowered women and girls about FP, the OR for this comparison would be different in direction to that of the overall OR for the community level. Moreover, in a 37.5% pairwise comparison, the odds of modern contraceptive utilization would be higher in the community with empowered women and girls about FP than in non-empowered. Further, in our model, the Median Odds ratio (MOR) was equal to 2.5 (empty model), 1.653 (individual factors), 2.194 (community factors), and 2.262 (combined model). Therefore, for the individual factors, the MOR was 2.5, whereas its reciprocal was 0.4. Therefore, examining the OR for individual factors in Table 4, four of the individual factors had an OR that lay outside of the interval (0.4, 2.5). Thus, the magnitude of the effect of clustering (cluster effect) was smaller than that of 4 of the seven individual factors. Therefore, the MOR of 2.5 is interpreted as fair because only 7.7% of the total variation in reproductive-age women's modern contraceptive utilization is due to cluster differences. Besides, MOR indicated that the unexplained community variation in the utilization of modern contraceptives reduced from 2.5(null model) to 2.26 (final model). This shows that when all predictors are considered, the effect of clustering is still statistically significant (Table 4).

Table 4: - Estimated variance components, a proportional change in variance, Shrinkage factor, interval odds ratio, proportion of opposed odds ratio, the median odds ratio of Ethiopia, PMA, 2019

| **Variable** |  | | | | | | |
| --- | --- | --- | --- | --- | --- | --- | --- |
|  | **Empty model** | **Individual Factors** | | **Community Factors** | | | **Combined Factors** |
|  |  | **IOR lower** | **IOR Upper** | **IOR lower** | **IOR Upper** | **POOR** |  |
| **Community-level factors** |  |  |  |  |  |  |  |
| Community way of life |  |  |  | 0.48 | 10.7 | 0.248 |  |
| Community literacy level |  |  |  | 0.308 | 6.87 | 0.378 |  |
| women and girls' empowerment about FP |  |  |  | 0.31 | 6.927 | 0.375 |  |
| Community attitude/norms towards FP |  |  |  | 0.29 | 6.46 | 0.397 |  |
| Variance (τ ^2)^ | 0.928 | 0.278 |  | 0.679 | | | 0.732 |
| Shrinkage factor | 0.893 | 0.955 |  | 0.899 | | | 0.893 |
| VPC/ICC | 0.219 | 0.077 |  | 0.171 | | | 0.182 |
| MOR | 2.5 | 1.653 |  | 2.194 | | | 2.262 |
| PCV | Ref | 69.8% |  | 26.4% | | | 20.5% |
| AIC | 7391.5 | 3366.9 |  | 7319.0 | | | 4295.42 |

AIC: Akaike Information Criterion, VPC: variance partition coefficient, ICC: Intra-class correlation, PCV: proportional change of the variance, IOR: Interval odds ratio, POOR: the proportion of odds ratios in the opposite direction, MOR: median odds ratio

## Individual and community-level determinants of utilization of Modern contraceptives

### Effect of individual-level characteristics on modern contraceptive use

After adjusting for the individual and community factors, reproductive-age women with the marital status of divorced/widowed were less likely to report modern contraceptive use (AOR:0.18, 95% CI 0.13,0.23) than never-married women. The data also showed that the odds ratio of modern contraceptive use increases with an increasing level of education. For instance, women who attended primary school, secondary school, and more than secondary were more likely to report modern contraceptive use (AOR: 1.47, 95% CI 1.20, 1.79), (AOR: 1.73, 95% CI 1.33, 2.26) and (AOR: 1.58, 95% CI 1.16, 2.17), respectively than with no education. Moreover, women who reported having facility visits for family planning discussions in the last 12 months were more likely to report modern contraceptive use (AOR:1.84, 95% CI 1.52, 2.23) compared to never having had a visit in the last 12 months (Table 5).

### Effect of community-level factors on modern contraceptive use

Accordingly, after controlling the effect of individual and community factors, clusters with a high number of agrarian community ways of life were reported to have better modern contraceptive use (AOR: 2.27, 95% CI 1.5, 3.44), and higher community literacy levels were reported to have better modern contraceptive use (AOR: 1.46, 95% CI 1.09, 1.94). Similarly, clusters that empowered women and girls about FP were more likely to report FP use (AOR: 1.47, 95% CI 1.11, 1.93) than non-empowered clusters. Besides, clusters with supportive community attitudes/norms toward FP are associated with higher odds of modern contraceptive use (AOR: 1.37, 95% CI 1.04, 1.81) than clusters with non-supportive community attitudes/norms towards FP (Table 5).

Table 5: - Estimated odds ratios for multilevel logistic regression model of modern contraceptive use of respondents, Ethiopia, PMA, 2019

| **Variable** | **Odds ratio (95% CI)** | | | |
| --- | --- | --- | --- | --- |
|  | **Empty model** | **Individual-level factors** | **Community-level factors** | **Combined factors** |
| **Individual-level factors** |  |  |  |  |
| Birth events |  | 0.91(0.82, 1.01) |  |  |
| Marital status: Divorced/widowed |  | 0.2(0.15, 0.27) *** |  | 0.18(0.13,0.23) *** |
| School Attended: Primary |  | 1.25(1, 1.56) * |  | 1.47(1.20,1.79) *** |
| School Attended: Secondary |  | 1. 36(1.01,1.81) * |  | 1.73(1.33,2.26) *** |
| School Attended: More than secondary |  | 1.03 (0.75, 1.42) |  | 1.58(1.16,2.17) ** |
| Marriage history: More than once |  | 0.73 (0.58, 0.92) ** |  | 0.89(0.71,1.11) |
| Visited by a health worker |  | 1.25(0.97,1.61) |  |  |
| Visited group counseling |  | 1.89(0.67,1.17) |  |  |
| Facility FP discussion |  | 1.6(1.29,1.97) |  | 1.84(1.52,2.23) *** |
| **Community-level factors** |  |  |  |  |
| Community knowledge of the modern contraceptive method |  |  | 1.02(0.76,1.99) |  |
| Community attitude/norms towards FP |  |  | 1.39((1.09,1.78) ** | 1.37(1.04,1.81) * |
| Women and girls empowerment about FP |  |  | 1.53(1.2,1.95) *** | 1.47(1.11,1.93) ** |
| Exposure to family planning information |  |  | 0.96(0.7,1. 32) |  |
| Community way of life |  |  | 2.55(1.77, 3.68) *** | 2.27(1.5, 3.44) *** |
| Community literacy level |  |  | 1.62(1.22,2.16) *** | 1.46(1.09,1.94) ** |

Significant at p-value 0 ‘***’ 0.001 ‘**’ 0.01 ‘*’ 0.05,

FP: Family Planning

# Discussion

Modern contraceptive use in this current study is indicated to be influenced by individual-level factors such as the marital status, education status of women, and women’s family planning discussion at the health facility. From the community level factors, clusters having supportive norms about family planning, higher women and girls’ empowerment about family planning, being agrarian as a community way of life, and a higher community level of literacy were found to be significantly associated with increased modern contraceptive use compared to clusters with lower reports of these characteristics. Individual- and community-level factors simultaneously were responsible for approximately 20.5% of the modern contraceptive use among reproductive-age women in Ethiopia. The findings of this study will be generalized to all reproductive age women in Ethiopia.

This study showed that the prevalence of modern contraceptive use was low, with only one-third of reproductive-age women using a modern contraceptive at the time of the survey. This aligns with the Ethiopian demographic and health survey 2016 results (30). However, it is higher than the demographic and health surveys result in Sub-Saharan Africa (31). Hence, it must be noted that this proportion of use was very low to achieve and meet Ethiopia's global family planning 2020 (FP2020) targets, which were to increase CPR to 55% and TFR to three (9). For instance, Ethiopia was behind the ambitious goals and targets of the FP2020. Ensuring the FP2020 goal was a critical milestone in achieving the goals and targets of HSTP-II and Sustainable Development Goals (SDGs), target 3.7, which calls on countries “by 2030, to ensure access to family planning, information, and education. Though much progress has been made, there is still long way to go to attain the country’s family planning goals. Therefore, further effort is needed to put in place and enhance the use of modern contraceptives among reproductive-age women in Ethiopia.

Concerning reproductive-age women’s marital status, in this study, women who never married had a better chance of using modern contraceptives than those who were divorced/widowed and currently married. Similarly, a study indicated that in sub-Saharan Africa, overall contraceptive prevalence appears to be higher among never-married women than currently married women. Currently, married women usually have the lowest contraceptive prevalence (32). Moreover, a study on contraception in adolescence: the influence of parity and marital status on contraceptive use in 73 low and middle-income countries indicated that married female adolescents have the lowest median modern contraceptive prevalence in all world regions (33). The possible justification might be that divorced / widowed women might not have attained the desired number of children and infrequent sexual intercourse, making them less likely to use contraceptives. The overall result implies that being married-women might not pledge the women to use a contraceptive. So, family planning interventions have to focus on all reproductive age women rather than narrowed to married women only.

We found that the odds ratio of modern contraceptive use increases with an increasing level of education up to secondary education. However, a bit of a decline in contraceptive use among women with above secondary education compared with secondary education was indicated. A study done in Nigeria indicated that increasing levels of education increase modern contraceptive use (34). Many studies also show that a woman's educational attainment is positively associated with contraception use (35–37). Moreover, education alone was indicated to have had a significant effect on the use of contraception (38). Women were less likely to use modern contraceptives without education (31). The possible reason for the decrement in modern contraceptive use in those above secondary education might be due to late marriage after the completion of higher education and the desire for more children.

Having family planning discussions at a health facility was positively associated with modern contraceptive use. This is in line with a study done in Uganda discussing FP with a field or health worker that remained statistically significant (39). Women were less likely to use modern contraceptives if they were not told of family planning at a health facility (31). Therefore, as women's contact with health providers increases, the probability of obtaining information and contraceptive use will be improved. Thus, the women who get an excellent opportunity to the awareness of FP would be vital to fill the missed opportunity (30). In addition, it creates a conducive environment that helps mitigate the myths and misconceptions about FP and enhances the relationship between the health care providers and women, thereby increasing FP use. Such collective effort would contribute to increasing women's actual modern contraceptive use.

Community norms/attitude towards FP use was a cluster-level factor influencing contraceptive use. Our finding aligns with another study done in Ethiopia and 73 low-and middle-income countries (33,40). In this study, women from clusters with supportive attitudes/norms toward family planning were associated with FP use. Moreover, another study revealed that social pressure on women to bear and not bear children incurs social disapproval (41). So, this was indicated to have a major influence on the intentions and behaviors of women regarding spacing and contraceptive use. Hence, it is noted that effort should be put into overcoming and changing the community attitude/norms regarding FP with the focus of promoting FP use may encourage women to use contraceptives.

This study indicates that higher women and girls’ empowerment in family planning was positively associated with modern contraceptive use. It was also indicated from the community-level variables that women who reside in a cluster with lower women and girls’ empowerment about family planning had lower odds of contraceptive use. These findings align with a study in Ethiopia revealing women’s empowerment as an essential determinant of contraceptive use. Moreover, empowered women were more likely to use contraceptives than un-empowered women (42). Similarly, another study in Tanzania indicated that self-efficacy for contraception was associated with using modern contraceptives (35). Women who had complete control over their healthcare were more likely to use contraceptives than women who had no control over their healthcare (34). A systematic review also revealed that women’s empowerment domains are consistently positively associated with contraceptive ever use. In many Sub-Saharan African countries, this could also be justified as women’s roles and reproductive choices could be constrained by various cultural traditions that restrict women’s autonomy and encourage men’s dominance (43). This disempowering of women and girls might directly impact women’s decision to use contraceptives.

Consistent with previous studies, this study also revealed a positive association between a community literacy level and modern contraceptive use. Cluster with higher women’s literacy status at the community level was positively associated with using modern contraceptives. Pieces of evidence from many individual studies also indicated that women from the community with higher women’s literacy status were more likely to be users of contraceptives than their reference group. Generally, women tend to get higher decision-making power about contraceptives by attaining higher literacy levels (40,44–47).

In addition, It has been argued that being literate has a potential role in shaping individuals’ perceptions and knowledge about modern contraceptives, which helps them overcome misconceptions about modern contraception use (37)**.** The current study indicated that many women could not read and write simple sentences. So, it is justified that being illiterate might make women unaware of the benefit of the optimal birth interval and could not overcome the misconceptions about contraceptive use. This might make clusters with low community literacy levels consider giving birth with a narrow birth interval as normal. The effect of community literacy in disseminating contraceptive information and counseling women is profound. The cumulative effect brings or predicts the actual use of contraceptives. This implies that a particular effort is needed to increase the number of women who are literate and, thereby, intend to use contraceptives.

Our study found clusters of being agrarian as a community way of life positively associated with modern contraceptive use. This is in line with studies done in the Afar region. According to a study in the Afar region, the influence of the husband, religious, and clan leader regarding FP might lead to low modern contraceptive use. In addition, the non-supportive attitude of the pastoralist community toward family planning methods and the women’s low educational status accompanied by low community literacy might end up in low contraceptive use (40,48). This might be because agrarian communities have better infrastructure, schools, health facilities and media access, and male involvement in FP decision, creating a more conducive environment for more contraceptive users than pastoralist community. Hence, family planning intervention programs should consider the community's way of life while intervening.

The study has both strengths and limitations. As a strength, it focused on a large sample size that included unmarried women, a population most often excluded or underrepresented in most studies of modern contraceptive use. In addition, a summary measure of the effect of cluster variables and measures of heterogeneity components was done besides the normal interpretation of multilevel analysis using odds ratio and PCV and VPC/ICC. However, the study focused on reported contraceptive use, which may suffer from recall bias. Furthermore, the study did not exhaust all possible factors influencing contraceptive use, as the data were restricted to a national study that focused instead on covering a broad range of family planning questions.

Conclusion: Modern contraceptive use among reproductive-age women in Ethiopia is influenced by individual and community factors. An effort must be made to educate reproductive-age women, focusing on modern contraceptive methods. Investing in the education of reproductive-age women and the community is crucial. Education programs should target empowering girls/women and create communities to have supportive attitudes/norms towards FP. Finally, we recommend that health workers use any opportunity to counsel women who come to health facilities about the role that FP plays in reducing maternal and perinatal morbidity and mortality and encourage them to enhance reproductive-age women’s attitudes and practice modern contraceptive methods.

# Acknowledgments

Our deepest gratitude and appreciation go to the performance monitoring for action (PMA-Ethiopia) program for allowing us to use these survey data sets.

# Funding: The funder has no role in the designing, analysis and interpretation of the results.

Abbreviations: CI, Confidence Interval; ICC, Intraclass Correlation Coefficient; IOR, Interval Odds Ratio; MOR, Median Odds Ratio; PCV, Proportional Change in Variance; PMA, Performance Monitoring for Action; POOR, Proportion of Odds Ratios in the Opposite Direction; VPC, Variance Partition Coefficient

# Authors contributions

**Conceptualization:** Hailay Gebremichael Gebrekidan, Gurmesa Tura Debelew

**Data Curation:** Hailay Gebremichael Gebrekidan

**Formal Analysis:** Hailay Gebremichael Gebrekidan, Mussie Alemayehu

**Supervision:** Mussie Alemayehu, Gurmesa Tura Debelew

**Validation:** Mussie Alemayehu, Gurmesa Tura Debelew

**Visualization:** Mussie Alemayehu, Gurmesa Tura Debelew

**Writing-original draft:** Hailay Gebremichael Gebrekidan

**Writing- review, and editing:** Hailay Gebremichael Gebrekidan, Mussie Alemayehu, Gurmesa Tura Debelew

ORCID id: Hailay Gebrekidan: <https://orcid.org/0000-0002-3129-1221>

# References

1.	United Nations. Report of the International Conference on Population and Development [Internet]. New York; 1994. Available from: https://www.unfpa.org/sites/default/files/event-pdf/icpd_eng_2.pdf

2.	Ahmed S, Li Q LLT. Maternal deaths averted by contraceptive use: An analysis of 172 countries. Lancet [Internet]. 2012;380:111–25. Available from: http://dx.doi.org/10.1016/S0140-6736(12)60478-4

3.	Seltzer JR. The origins and evolution of family planning in developin countries [Internet]. Santa Monica: RAND; 2002. Available from: https://www.rand.org/content/dam/rand/pubs/monograph_reports/2007/MR1276.pdf

4.	UNDESA. World Family Planning 2022, Meeting the changing needs for family planning: Contraceptive use by age and method [Internet]. United Nations. New York: United Nations, Department of Economic and Social Affairs, Population Division; 2022. 43 p. Available from: https://desapublications.un.org/file/1083/download

5.	Cleland J, Bernstein S, Ezeh A, Faundes A GAI. Family planning: the unfinished agenda. Lancet. 2006;368(9549):1810–27.

6.	EFMoH E&. Ethiopia Mini Demographic and Health Survey 2019: Final Report [Internet]. Addis Ababa, Ethiopia; 2019. Available from: https://dhsprogram.com/pubs/pdf/FR363/FR363.pdf

7.	Ethiopia FMoH. Health Sector Transformation Plan II. Addis Ababa, Ethiopia; 2021.

8.	Bill & Melinda Gates Foundation & UNFPA. London Summit on Family Planning , July 2012. Technical Note: Data sources and methodology for developing the 2012 baseline, 2020 objective, impacts and costings (Working Draft - 15 June 2012) [Internet]. London, UK; 2012. Available from: https://fp2030.org/sites/default/files/2013_01-04_FP_Summit_technical_note_15_June.pdf

9.	FMoH. Costed Implementation Plan for Family Planning in Ethiopia 2015-2020 [Internet]. Addis Ababa, Ethiopia; 2016. Available from: http://www.healthpolicyplus.com/ns/pubs/2021-2030_EthiopiaCIPNov.pdf

10.	FP2020. FP2020 momentum at the midpoint 2015-2016 progress report [Internet]. New York; 2016. Available from: http://2015-2016progress.familyplanning2020.org/uploads/05/15/FP2020_DIGITAL_Spreads_LoRes.pdf

11.	Bearak J, Popinchalk A ALS. Global, regional, and subregional trends in unintended pregnancy and its outcomes from 1990 to 2014: estimates from a Bayesian hierarchical model. Lancet Glob Heal. 2018;6(4):e380–9.

12.	Singh S SGH. Unintended Pregnancy: Worldwide Levels, Trends, and Outcomes. Stud Fam Plann. 2010;41(4):241–50.

13.	Cleland J, Conde-Agudelo A, Peterson H RJT. Contraception and health. Lancet [Internet]. 2012;380(9837):149–56. Available from: http://dx.doi.org/10.1016/S0140-6736(12)60609-6

14.	Bongaarts, John, John C. Cleland, John Townsend, Jane T. Bertrand and MDG. Family Planning Programs for the 21 st Century Family Planning Programs for the 21 st Century [Internet]. New York; 2012. Available from: https://knowledgecommons.popcouncil.org/departments_sbsr-rh/1001/

15.	Guttmacher Institute. Adding It Up: Investing in Contraception and Maternal and Newborn Health in Ethiopia, 2018 [Internet]. Guttmacher Institute. New York; 2018. Available from: https://www.guttmacher.org/sites/default/files/factsheet/adding-it-up-contraception-mnh-ethiopia.pdf

16.	UKaid. London Summit on Family Planning: Summaries of Commitments [Internet]. London, UK; 2012. Available from: https://assets.publishing.service.gov.uk/government/uploads/system/uploads/attachment_data/file/67328/london-summit-family-planning-commitments.pdf

17.	UN. Transforming our World: The 2030 Agenda for sustainable development-Arsenic Research and Global Sustainability - Proceedings of the 6th International Congress on Arsenic in the Environment, AS 2016 [Internet]. Arsenic Research and Global Sustainability - Proceedings of the 6th International Congress on Arsenic in the Environment, AS 2016. Geneva, Switzerland; 2016. Available from: https://sustainabledevelopment.un.org/content/documents/21252030 Agenda for Sustainable Development web.pdf

18.	FP2020. FP2020 Measurement learning series: Measuring Contraceptive Use Among All Women: Advances and challenges [Internet]. 2020. Available from: https://fp2030.org/sites/default/files/Data-Hub/allwomen/MLS_allwomen_6_20.pdf

19.	Zimmerman L, Desta S, Mahari Y RAA etal. Protocol for PMA-Ethiopia : A new data source for cross- sectional and longitudinal data of reproductive , maternal , and newborn health [ version 1 ; peer review : 1 approved ]. Gates Open Res. 2020;4(126):1–16.

20.	V Ana RD. Residential Environments and Cardiovascular Risk. J Urban Heal Bull New York Acad Med. 2003;80(4):569–89.

21.	Merlo J, Ohlsson H, Lynch KF CBS. Individual and collective bodies : using measures of variance and association in contextual epidemiology. J Epidemiol Community Heal. 2009;63:1043–8.

22.	Merlo J FFF. Bringing the individual back to small-area variation studies : A multilevel analysis of all-cause mortality in Andalusia , Spain. Soc Sci Med. 2012;75:1477–87.

23.	Austin PC MJ. Intermediate and advanced topics in multilevel logistic regression analysis. In: Statistics in Medicine [Internet]. John Wiley & Sons Ltd; 2017. p. 3257–3277. Available from: https://pubmed.ncbi.nlm.nih.gov/28543517/

24.	Neuhaus JM MC. Separating between- and within-cluster covariate effects by using conditional and partitioning methods. JRoyal Stat Soc B. 2006;68:859–72.

25.	Larsen K MJ. Appropriate Assessment of Neighborhood Effects on Individual Health : Integrating Random and Fixed Effects in Multilevel Logistic Regression. Am J Epidemiol. 2005;161(1):81–8.

26.	Merlo J, Chaix B, Ohlsson H, Beckman A, Johnell K E etal. A brief conceptual tutorial of multilevel analysis in social epidemiology: using measures of clustering in multilevel logistic regression to investigate contextual phenomena. J Epidemiol Community Heal. 2006;60:290–7.

27.	Diehr P, Cain K CFV. What Is Too Much Variation ? The Null Hypothesis in Small-Area Analysis. Health Serv Res. 1990;24(6).

28.	Tessema ZT, Teshale AB, Tesema GA, Yeshaw Y WM. Pooled prevalence and determinants of modern contraceptive utilization in East Africa: A Multi-country Analysis of recent Demographic and Health Surveys. PLOSONE [Internet]. 2021;16(3):1–16. Available from: http://dx.doi.org/10.1371/journal.pone.0247992

29.	Merlo J, Wagner P GNL. An Original Stepwise Multilevel Logistic Regression Analysis of Discriminatory Accuracy: The Case of Neighbourhoods and Health. PLoS One. 2016;11(4).

30.	Central Statistical Agency (CSA) [Ethiopia] and I. Ethiopia demographic and health survey 2016 [Internet]. Addis Ababa, Ethiopia, and Rockville, Maryland, USA; 2016. Available from: https://dhsprogram.com/pubs/pdf/FR328/FR328.pdf

31.	Boadu I. Coverage and determinants of modern contraceptive use in sub-Saharan Africa: further analysis of demographic and health surveys. Reprod Health [Internet]. 2022;19(18). Available from: https://doi.org/10.1186/s12978-022-01332-x

32.	Wang, Wenjuan, Sarah Staveteig, Rebecca Winter and CA. Women’s marital status, contraceptive use, and unmet need in Sub-Saharan Africa, Latin America, and the Caribbean: DHS Comparative Report No. 44 [Internet]. Maryland, USA; 2017. Available from: http://dhsprogram.com/pubs/pdf/CR44/CR44.pdf

33.	Vargas C De, Coll N, Ewerling F, Hellwig F, Jardim A BDD. Contraception in adolescence : the influence of parity and marital status on contraceptive use in 73 low-and middle- income countries. Reprod Health. 2019;16(21):1–13.

34.	Solanke BL OPS. Women’S Empowerment As a Correlate of Contraceptive Use in Nigeria. J Res Natl Dev. 2014;12(1):120–31.

35.	Nsanya KM, Atchison CJ, Bottomley C DAK. Modern contraceptive use among sexually active women aged 15-19 years in North-Western Tanzania: Results from the Adolescent 360 (A360) baseline survey. BMJ Open. 2019;9(8).

36.	ME. P. Socio-economic and demographic factors affecting contraceptive use in Malawi. Afr J Reprod Health. 2013;17(3):91–104.

37.	Ahinkorah BO, Seidu AA, Appiah F BEA etal. Individual and community-level factors associated with modern contraceptive use among adolescent girls and young women in Ethiopia: a multilevel analysis of 2016 Ethiopia demographic and health survey. Contracept Reprod Med. 2020;5(27):1–12.

38.	Waiz NK. Role of education in the use of contraception. Lancet Perspect. 2000;356.

39.	Birabwa C, Chemonges D, Tetui M, Baroudi M NF etal. Knowledge and Information Exposure About Family Planning Among Women of Reproductive Age in Informal Settlements of Kira Municipality, Wakiso District, Uganda. Front Glob Women’s Heal. 2021;2(May).

40.	Mussie A, Lemma H, Kidan A AYF etal. Family planning use and associated factors among pastoralist community of afar region , Eastern Ethiopia. BMC Womens Health [Internet]. 2016;16(39):1–9. Available from: http://dx.doi.org/10.1186/s12905-016-0321-7

41.	Kane S, Kok M, Rial M, Matere A, Dieleman M BJ. Social norms and family planning decisions in South Sudan. BMC Public Health [Internet]. 2016;16(1183):1–12. Available from: http://dx.doi.org/10.1186/s12889-016-3839-6

42.	Muluneh MD, Francis L, Ayele M, Abebe S, Makonnen M S V. The Effect of Women ’ s Empowerment in the Utilisation of Family Planning in Western Ethiopia : A Structural Equation Modelling Approach. Int J Environ Res Public Heal. 2021;18(6550).

43.	Ndola P, Fraser A, Huchko MJ GJW etal. Women’s empowerment and family planning: A review of the literature. J Biosoc Sci. 2017;49(6):713–43.

44.	Wasswa R KAA. Multilevel mixed effects analysis of individual and community level factors associated with modern contraceptive use among married women in Uganda. BMC Public Health. 2021;21(1457):1–13.

45.	Mare KU, Aychiluhm SB TAM. Individual and community-level determinants of non-use of contraceptive among women with no fertility desire in Ethiopia: a multilevel mixed-effect analysis. Emerg Themes Epidemiol [Internet]. 2022;19(2):1–10. Available from: https://doi.org/10.1186/s12982-022-00112-z

46.	Mutumba M WES. Community influences on modern contraceptive use among young women in low and middle-income countries: A cross-sectional multi-country analysis. BMC Public Health. 2018;18(430):1–9.

47.	Hameed W, Azmat SK, Ali M, Sheikh MI AG et al. Women’s empowerment and contraceptive use: The role of independent versus couples’ decision-making, from a lower middle income country perspective. PLoS One. 2014;9(8).

48.	Alemayehu M, Medhanyie AA, Reed E et al. Individual-level and community-level factors associated with the family planning use among pastoralist community of Ethiopia: a community-based cross-sectional study. BMJ Open. 2020;10(9):e036519.

Figure 1: Selection of eligibility women for contraceptive method utilization among reproductive-age women in Ethiopia.
