# Supplementary material for: Individual and community level factors associated with modern contraceptive utilization among women in Ethiopia: Multilevel modeling analysis
Source: PLoS One. 2024 May 16;19(5):e0303803. doi: 10.1371/journal.pone.0303803 (PMC11098393; doi:10.1371/journal.pone.0303803)
Supplement: Attachment — Submitted filename: Response to Reviewers.docx [file pone.0303803.s004.docx]

**Date: Dec 1/2023**

**Manuscript Number: PONE-D-23-09872**

**Title:** Individual and community level factors associated with modern contraceptive use among women in Ethiopia: Multilevel modeling analysis

By: Hailay Gebremichael Gebrekidan^1,2*^, Mussie Alemayehu^2^, Gurmesa Tura Debelew^1^

To: Plos one

Dear Editor,

We, the authors of this manuscript, are pleased to thank the journal editors and the reviewers for the in-depth review of the manuscript and for giving us valuable comments and suggestions that helped us improve the manuscript. **The authors received no specific funding for this work.** We have rigorously revised the manuscript as per your questions and comments. We have included the point-by-point response in the table below, framed as reviewers' comments/questions and authors' responses. The detailed revisions and changes we made in the main document are prepared with track changes attached separately. We expect our revision will enable the manuscript to fit the journal better. If you have any additional questions, please do not hesitate to contact us anytime.

**Point-by-point Responses to Reviewers and Editor's Comments**

| **Reviewers' comments/questions** | Authors Response |
| --- | --- |
| **Editor** |  |
| 1. Regarding financial disclosure   The grant information you provided in the "Funding information" and "Financial disclosure" sections does not match | **Thank you very much, Editor**  The authors received no specific funding for this work, and It is revised and corrected in the manuscript under the funding information section. |
| 2. Data availability statement:   -A description of the data set and the third-party source - If applicable, verification of permission to use the data set - Confirmation of whether the authors received any special privileges in accessing the data that other researchers would not have - All necessary, non-author contact information, URLs, etc., others would need to apply to gain access to the data. | We used a cross-sectional dataset from the Performance Monitoring for Action Ethiopia (PMA-ET) 2019 survey, accessed after submitting our study's abstract to PMA-Ethiopia at <https://www.pmadata.org/countries/ethiopia>. The terms and conditions of the PMA-Ethiopia data do not allow sharing the data with others (only used for the registered data). We didn't receive any special privileges in accessing the data. Thus, any researcher can apply to access the data from the PMA-Ethiopia office at <https://www.pmadata.org/countries/ethiopia> using the details described in this paper's Methods and Materials section.  It is revised and corrected as per the comments. |
|  | **Finally, we, the authors, are very grateful to you for taking your precious time and significantly contributing to improving the manuscript.** |
| **Reviewer #1:** |  |
| **Abstract** |  |
| 1. Your abstract too shallow especially in background and conclusion part. | **Thank you very much, reviewer**  We accepted your comments and replaced them with more comprehensive words and evidence. |
| 2. What about other finding and factors associated with modern contraceptive methods like socio-demographic status, fertility status. | **Thank you again for your comment**  The individual-level factors are considered as the socio-demographic factors. We added the fertility-related result in Table 1. |
| 3. Your finding is not matched with your study protocol which it is minimized | **Thank you very much, reviewer**  It is revised and corrected as per the comments and suggestions. |
| 4. Your conclusion in the abstract is not based on your findings | **Thank you for your valuable comment**  It is revised as requested |
| **Back ground** |  |
| 1. Your background is too wide you should have minimized based on journal guidelines | **Thank you very much, reviewer**  We accepted your comments and revised them as requested |
| 2. In paragraph 1 you have explained only married women what about unmarried but uses family planning based on your title. | **Thank you, dear reviewer, for your valuable contribution**  We accepted your comments and revised them as requested |
| 3. Your first paragraph should be defined the problem or subject matter rather than general term. | **Thanks again, reviewer**  It is revised as per the comment |
| 4. Then the next paragraphs start to problem-based idea and its prevalence, burden, consequence, strategies to overcome and complication from global to local | **Thank you very much, reviewer**  We accepted your comments and included other more comprehensive evidence per the comment. |
| 5. Your background not talk about general facts of your study outcome variables and to shallow, add as comment given to in your document. | **Thank you, dear reviewer**  We accepted your comments and included other more comprehensive evidence per the comment. |
| 6. Your back ground amalgamated terminologies and it needs revision one by one. | **Thanks again, dear reviewer**  It is revised as per the comment |
| 7. Most of your document lacks the references and need updating at least five year duration. | **Thank you very much, reviewer**  It is revised as per the comment, and referencing was managed accordingly |
| 8. If you are using secondary data, you don't mention about data extraction period. | **Thank you very much, reviewer**  We used secondary data from performance monitoring for action Ethiopia (PMA-Ethiopia) 2019 survey data. So, we are referring to the data point of the cross-sectional 2019 data. |
| 9. Some of idea in your document it lacks clarity, typographical errors especially grammar how did you access it. | **Thank you very much, reviewer**  We would like to thank the reviewer for the valuable and constructive comments. |
| 10. Justification /rationale of the study is not clear. You have to clearly state why this study was necessary in concise and coherent way | **Thank you very much, reviewer**  We accepted your comments and included other more comprehensive evidence per the comment. |
| **Methods:** |  |
| 1. How you see the difference of study population and sampled population? Correct it. | **Thank you, dear reviewer, for your question**  All reproductive-age women were considered the study population, whereas the reproductive-age women included in the sample were considered the sampled population. So, it was corrected accordingly. |
| 2. Focus only on the objective of the study. | **Thank you very much, reviewer**  It is corrected accordingly. |
| 3. EDHS 2019 was mini-EDHS and have you accessed all variables what you want to analyze? | **Thank you again, dear reviewer, for your question**  We used performance monitoring for action Ethiopia (PMA-ET 2019) cross-sectional data. We do not use EDHS 2019 for analysis. We only used it as a reference material. |
| 4. In Ethiopia each region had multi diversified populations, cultural, political, religious etc. how did you justified the sampling methods and stratification methods urban or rural, large region or small region. It is difficult to estimate and generalize based on your way of interpretation and sampling methods. | **Thank you dear reviewer for your suggestion** PMA-ET was conducted in two stages; first, each region was stratified into urban and rural areas to form sampling strata. Then, samples of enumeration areas (EAs) were selected independently in each stratum in the second stage. However, the current analysis is based on the secondary data accessed from the PMA-ET 2019 data. Hence, the details about the sampling procedure are available at https://www.pmadata.org/countries/ethiopia. |
| 5. Inclusion and exclusion criteria, dependent variable and independent variables should be clearly justified. | **Thank you very much for your valuable suggestion.**  It is revised as per the comment. |
| 6. What are the unknown confounding factors of this study finding and think your study design? | **Thank you, dear reviewer, for your question**  During the design and sampling, PMA-Ethiopia used stratification techniques to sample enumeration areas from urban/rural strata and large and small regions to achieve the best representative sample and control the unknown confounding factors.  This study used a multilevel logistic regression analysis to account for the unknown confounders. So, first, we organize the data in a hierarchical structure. We specify the level at which each predictor variable should be included (individual level or community level), add all variables as predictors in the model, assess the model fit and significance, and interpret the adjusted odds ratios and associated confidence intervals. |
| **Result** |  |
| 1. How did you categorized age category, why not based on WHO category or EDHS itself category? Is it inclusive? Because majority of rural part of community doesn't know their real date of birth | **Thank you very much, reviewer**  Based on other related literature, we put the age category to easily understand for readers the advantage of age for maternal health. The age group of 15- 19 years is labeled as "young maternal age," the age group of 20-34 years is labeled as "adult maternal age," and those greater than 35 years of age are older maternal age. The experience of contraceptive practice, healthy mother, pregnancy, and babies have a direct relationship with the age of mothers. That is why we prefer to use the stated age category over the WHO/EDHS age category. |
| 2. Please carefully cross-check the numbers (percentages) in the texts and respective tables (E.g. Table 1(No education 2600 but Literacy for those with no education (2589), wealth index, total live birth etc.), please revise and put denominator in each part of table | **Thank you very much, reviewer**  It is corrected accordingly, and we put a denominator in each part of the table heading. |
| 3. Is it homogenies which means cluster? What are non-cluster parts of study populations? | **Thank you very much, reviewer**  In PMA-ET, the word cluster is to mean Enumeration area (EA). EA is the primary sampling unit in the PMA-ET sampling period. During the 2019 PMA-ET survey, they had a total of 265 EAs, and from each EA, 35 households were randomly selected for the household and eligible female (15-49 years of age) interview. So, all the study populations were parts of a certain cluster (EA). In this study, we used the words cluster and EA interchangeably throughout the document. |
| **Discussion** |  |
| 1. Your discussion part should be rewritten. Please focus only in line with your important findings and objectives of the study. Factors like the marital status, education status of women is it analyzed and output of your study? | **Thank you very much, reviewer**  It is revised accordingly. Yes, both marital status and education status were analyzed and found to be significant predictors of modern contraceptive use. |
| 2. Your first paragraph should be your magnitude and objective of your study then factors associated with. | **Thank you very much, reviewer**  It is revised as per the comment |
| 3. Recommendation and strong conclusion should be needed | **Thank you very much, reviewer**  We accepted your comments, revised it, and put a strong conclusion per the comment. |
| 4. Limitation of the study needed | **Thank you very much, reviewer**  It is revised as per the comment |
|  | **Finally, we, the authors, are very grateful to you for taking your precious time and significantly contributing to improving the manuscript.** |
|  |  |
| **Reviewer # 2** |  |
| 1. The problem statement that led to this study is not clear. FP is key in reducing maternal and perinatal morbidity and mortality and induced abortion related maternal deaths. This is not clearly stated | **Thank you very much dear reviewer for your valuable comment**  These statements have been revised, and some confusing ideas have been deleted. |
| 2. The document may have been corrupted when I downloaded it. However there seems to be a cut and paste of irrelevant material on pesticides? It was a bit confusing to find this within the manuscript | **Thank you very much, reviewer**  These statements have been revised, and some confusing ideas have been deleted. |
| 3. The manuscript looked very long and can be summarized especially the discussion for the findings of the three objectives | **Thank you very much, reviewer**  It is revised as per the comment |
| 4. Clearly indicate the new knowledge that this study provides on prevalence and factors influencing contraceptive use in Ethiopia | **Thank you very much, reviewer**  It is revised as per the comment |
| 5. The data set used and the sample size is appropriate. However, indicate how the women consented to participate in this study as stated. Or does the author mean women consented to participate in the PMA? Clarify | **Thank you dear reviewer for your question**  Consent was not required for this study since we have used publicly available PMA-Ethiopia datasets. The data were accessed from http//[www.pmadata.org/countries/ethiopia](http://www.pmadata.org/countries/ethiopia) after the purpose of the study was explained, and the data-sharing policy was followed. But, PMA-ET has received the consent of each respondent during the PMA-ET 2019 survey period. |
| Line 15, delete the word "current" | **Thank you so much, dear, for your comment**  We deleted the word "current" and replaced it with "The prevalence of." |
| Line 17, "information exchange" do you mean awareness of family planning? | **Thank you so much, dear, for your comment**  "Information exchange" is replaced by "Discussion about family planning between women and health care providers." |
| Line 42, We are talking about sustainable development goals. Not MDGs. Please revise | **Thank you very much, reviewer**  It is revised accordingly |
| Line 77, provide citation of these studies |  |
| Line 108, replace the word "is" by "was" | **Thank you so much, dear, for your comment**  We replaced the word as per your comment |
| Line 359, "reference number 30" sorry could be my computer that corrupted this document. But this section does not make sense | **Thank you very much, reviewer**  It is revised per the comment and might be visible in the revised version. |
| Line 398, "reference number 30" This does not make sense on this manuscript. It looks corrupted | **Thank you very much, reviewer**  The revised version might be visible |
| Page 22, "References" I was not able to follow the reference list. Looks corrupted for reasons I cannot understand | **Thank you very much, reviewer**  The reference might be visible in the revised version. |
|  | **Finally, we, the authors, are very grateful to you for taking your precious time and significantly contributing to improving the manuscript.** |

Sincerely,

Hailay Gebremichael Gebrekidan (On behalf of the authors)

Jimma University, Department of reproductive and family health

Tell: +251966346756	email: [hailaygeb23@gmail.com](mailto:hailaygeb23@gmail.com)
