# Supplementary material for: Individual and community level factors associated with modern contraceptive utilization among women in Ethiopia: Multilevel modeling analysis
Source: PLoS One. 2024 May 16;19(5):e0303803. doi: 10.1371/journal.pone.0303803 (PMC11098393; doi:10.1371/journal.pone.0303803)
Supplement: Attachment — Submitted filename: Response to Reviewers.docx [file pone.0303803.s005.docx]

**Date: March 8/2024**

**Manuscript Number:** PONE-D-23-09872R2

**Title:** Individual and community level factors associated with modern contraceptive utilization among women in Ethiopia: Multilevel modeling analysis

By: Hailay Gebremichael Gebrekidan^1,2*^, Mussie Alemayehu^2^, Gurmesa Tura Debelew^1^

To: Plos one

Dear Editor and reviewers

We, the authors of this manuscript, are very grateful for the valuable comments and suggestions you gave us to improve the paper. Hereunder, we responded to each point raised in the second-round review, and the changes are indicated in the track change section of the revised manuscript. We have rigorously revised the manuscript based on your questions and comments. We have included the point-by-point response in the table below, framed as reviewers' comments/questions and authors' responses. The detailed revisions and changes we made in the main document are prepared with track changes attached separately. We expect our revision will enable the manuscript to fit the journal better. If you have any additional questions, please do not hesitate to contact us anytime.

**Point-by-point Responses to Reviewers and Editor's Comments**

| **Reviewers' comments/questions** | Authors Response |
| --- | --- |
| **Editor** |  |
| **Journal requirements** |  |
| 1. Please review your reference list to ensure that it is complete and correct. If you have cited papers that have been retracted, please include the rationale for doing so in the manuscript text, or remove these references and replace them with relevant current references. Any changes to the reference list should be mentioned in the rebuttal letter that accompanies your revised manuscript. If you need to cite a retracted article, indicate the article's retracted status in the References list and also include a citation and full reference for the retraction notice | **Thank you, very much dear Editor, for your suggestion.**  We have revised the reference list per your suggestion. |
| 2. We note that the grant information you provided in the 'Funding Information' and 'Financial Disclosure' sections do not match.  When you resubmit, please ensure that you provide the correct grant numbers for the awards you received for your study in the 'Funding Information' section. | **Thank you, very much dear Editor, for your question.**  We did not change financial disclosure as "the authors received no specific funding for this work." |
| 3. Thank you for stating the following financial disclosure:  "The authors received no specific funding for this work."  At this time, please address the following queries: A) Please clarify the sources of funding (financial or material support) for your study. List the grants or organizations that supported your study, including funding received from your institution. B) State what role the funders took in the study. If the funders had no role in your study, please state: "The funders had no role in study design, data collection and analysis, decision to publish, or preparation of the manuscript." C) If any authors received a salary from any of your funders, please state which authors and which funders. D) If you did not receive any funding for this study, please state: "The authors received no specific funding for this work."  Please include your amended statements within your cover letter; we will change the online submission form on your behalf. | **Thank you, dear Editor, for your question.**  We used a publicly available cross-sectional dataset from the Performance Monitoring for Action Ethiopia (PMA-ET) 2019 survey.   A) No financial or material support was received from any organizations, including our own institutions. B) Since the authors did not receive any funding, the funding statement was updated as "The authors received no specific funding for this work. The funders had no role in study design, data collection and analysis, decision to publish, or preparation of the manuscript." C) None of the authors of this manuscript has received any salary from the funders. D) Since the authors did not receive any funding, we updated it as "the authors received no specific funding for this work." |
| 4. Thank you for stating the following Funding Information in your manuscript:  "The authors received no funding for this work. Funders had no role in the design, analysis, interpretation of the results, or manuscript preparation. "  We note that you have provided funding information that is currently declared in your Funding Statement. However, funding information should not appear in any areas of your manuscript. We will only publish funding information present in the Funding Statement section of the online submission form.  Please remove any funding-related text from the manuscript and let us know how you would like to update your Funding Statement. Currently, your Funding Statement reads as follows:  "The authors received no specific funding for this work."  Please include your amended statements within your cover letter; we will change the online submission form on your behalf. | **Thank you for your suggestion.**  We have removed the funding-related text we provided in the manuscript. |
|  | **Finally, we, the authors, are very grateful to you for taking your precious time and significantly contributing to improving the manuscript.** |
| **Reviewer #1:** |  |
|  |  |
| 1. Language part and punctuation should be considered, and a few words highlighted and red-marked | **Thank you very much, reviewer**  We have reviewed and edited the document for grammar and punctuation errors. |
| 2. The background part is still wide if you can do so modify it | **Thank you again for your comment**  We have revised it per the comment |
|  |  |
| **Reviewer # 2** |  |
| 1. Conclusion on predictors of contraceptive uptake - 'The effect of clustering in modern contraceptive use was large compared to the unexplained between cluster variation and the individual-level factors' in the concluding statement does not make sense and needs to be re- written in line with study objectives | **Thank you, very much dear reviewer, for your valuable comment**  The statements have been revised to emphasize the study objectives, and any confusing ideas have been removed. In addition, we have incorporated the weighted demographic and prevalence results in Table 1 and the text of the main document. |
|  | **Finally, we, the authors, are very grateful to you for taking your precious time and significantly contributing to improving the manuscript.** |

Sincerely,

Hailay Gebremichael Gebrekidan (On behalf of the authors)

Jimma University, Department of reproductive and family health

Tell: +251966346756	email: [hailaygeb23@gmail.com](mailto:hailaygeb23@gmail.com)
