# Supplementary material for: Individual and community level factors associated with modern contraceptive utilization among women in Ethiopia: Multilevel modeling analysis
Source: PLoS One. 2024 May 16;19(5):e0303803. doi: 10.1371/journal.pone.0303803 (PMC11098393; doi:10.1371/journal.pone.0303803)
Supplement: Attachment — Submitted filename: Response to Reviewers.docx [file pone.0303803.s006.docx]

**Date: April 27/2024**

**Manuscript Number:** PONE-D-23-09872R2

**Title:** Individual and community level factors associated with modern contraceptive utilization among women in Ethiopia: Multilevel modeling analysis

By: Hailay Gebremichael Gebrekidan^1,2*^, Mussie Alemayehu^2^, Gurmesa Tura Debelew^1^

To: Plos one

Dear Editor and reviewers

We, the authors of this manuscript, are very grateful for the valuable comments and suggestions you gave us to improve the paper. Hereunder, we responded to each point raised in the third-round review, and the changes are indicated in the track change section of the revised manuscript. We have rigorously revised the manuscript based on your questions and comments. We have included the point-by-point response in the table below, framed as reviewers' comments/questions and authors' responses. The detailed revisions and changes we made in the main document are prepared with track changes attached separately. We expect our revision will enable the manuscript to fit the journal better. If you have any additional questions, please do not hesitate to contact us anytime.

**Point-by-point Responses to Reviewers and Editor's Comments**

| **Reviewers' comments/questions** | Authors Response |
| --- | --- |
| **Editor** |  |
| **Journal requirements** |  |
|  | **Finally, we, the authors, are very grateful to you for taking your precious time and significantly contributing to improving the manuscript.** |
| Reviewer #2: Lydia Mwanzia PhD Moi University, college of health sciences, Eldoret Kenya |  |
|  |  |
| 1. Include the following abbreviations in Acronym list- HHQ, FRS, SNNP, ODK | **Thank you very much, reviewer**  As per the comment, we have reviewed and included the remaining lists of abbreviations in the document. |
| 2. Abstract indicates the use of R software version 4.1 for analysis while the text indicate use of Stata Version 16.1. Which is which? Please clarify | **Thank you again for your comment**  We have revised it per the comment.  In Stata version 16.0, the data underwent cleaning, aggregation, and survey weighting, following which a descriptive analysis was performed utilizing the "svy" command. Subsequently, the main analysis was executed using R software version 4.1.3 |
| 3. The study findings add to the knowledge on need for Contraceptive programs that target pastoralist reproductive women | **Thank you, very much dear reviewer, for your valuable comment.**  We have revised it per the comment |
| 4. The literature review and discussion sections have long paragraphs in pros. The authors would improve this by adding some sub-headers to enhance readability. | **Thank you again for your comment**  After incorporating the feedback provided, we have revised the background and discussion sections accordingly. The paragraphs have been adjusted, and line space were added to indicate similar contents. While we have enhanced readability, we were unable to include subheadings in each paragraph due to our commitment to aligning the document with the formatting requirements outlined by the PLOS One journal guidelines. |
|  | **Finally, we, the authors, are very grateful to you for taking your precious time and significantly contributing to improving the manuscript.** |

Sincerely,

Hailay Gebremichael Gebrekidan (On behalf of the authors)

Tell: +251966346756	email: [hailaygeb23@gmail.com](mailto:hailaygeb23@gmail.com)
